# Supplementary material for: Circulation of Bluetongue Virus Serotypes 1, 4, 8, 10 and 16 and Epizootic Hemorrhagic Disease Virus in the Sultanate of Oman in 2020–2021
Source: Viruses. 2023 May 27;15(6):1259. doi: 10.3390/v15061259 (PMC10305157; doi:10.3390/v15061259)
Supplement: Supplementary file 1 [file viruses-15-01259-s001.zip › Oman viruses 230514.pdf]

Supplementary data S1. Phylogenetic analysis of BTV segments 1, 3, 4, 5, 7, 8, 9 and 10.

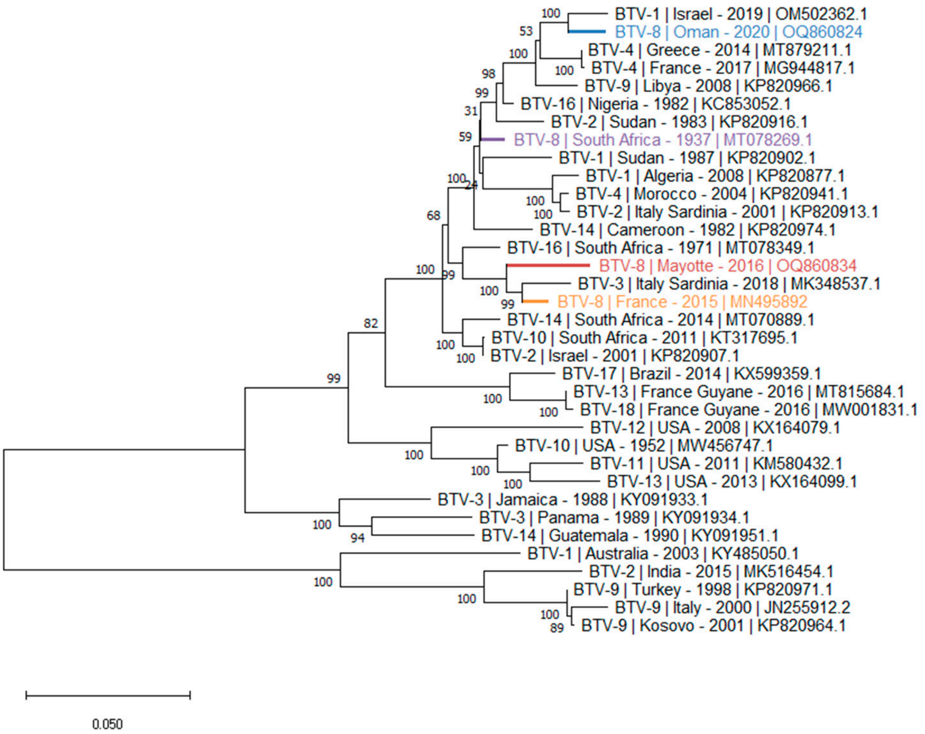

Figure S1: Phylogenetic analysis of BTV S1 sequences. Phylogenetic analysis of S1 sequences of BTV strains using the Maximum Likelihood method and Tamura-Nei model (1000 replicates). This analysis involved 35 nucleotide sequences, there were a total of 3906 positions in the final dataset. In the phylogenetic tree, GenBank sequences, species designations and strain names are given. The sequences investigated in the present study are marked in blue, red, orange and purple.

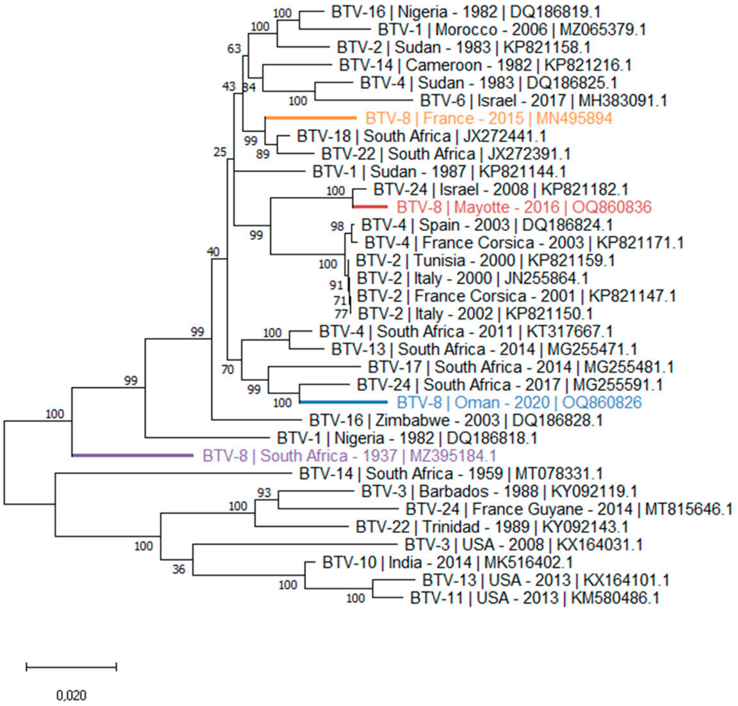

Figure S2: Phylogenetic analysis of BTV S3 sequences. Phylogenetic analysis of S3 sequences of BTV strains using the Maximum Likelihood method and Tamura-Nei model (1000 replicates). This analysis involved 34 nucleotide sequences,

there were a total of 2710 positions in the final dataset. In the phylogenetic tree, GenBank sequences, species designations and strain names are given. The sequences investigated in the present study are marked in blue, red, orange and purple.

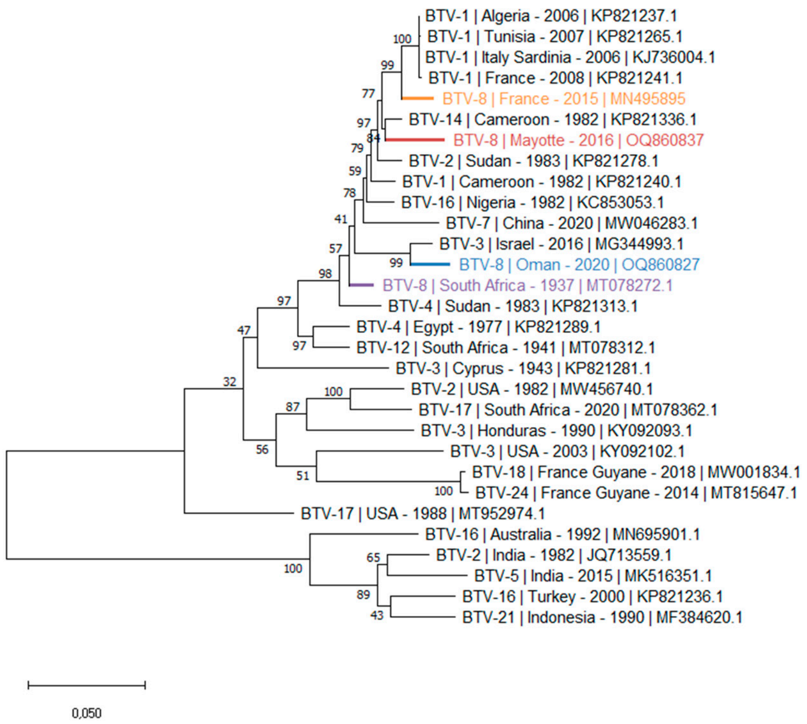

Figure S3: Phylogenetic analysis of BTV S4 sequences. Phylogenetic analysis of S4 sequences of BTV strains using the Maximum Likelihood method and Tamura-Nei model (1000 replicates). This analysis involved 30 nucleotide sequences, there were a total of 1935 positions in the final dataset. In the phylogenetic tree, GenBank sequences, species designations and strain names are given. The sequences investigated in the present study are marked in blue, red, orange and purple.

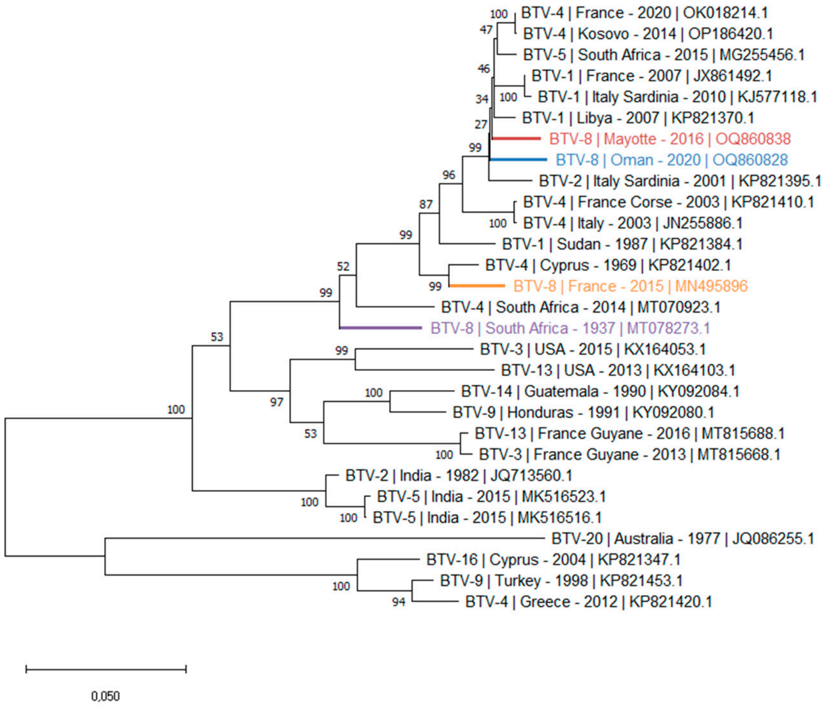

Figure S4: Phylogenetic analysis of BTV S5 sequences. Phylogenetic analysis of S5 sequences of BTV strains using the Maximum Likelihood method and Tamura-Nei model (1000 replicates). This analysis involved 29 nucleotide sequences, there were a total of 1773 positions in the final dataset. In the phylogenetic tree, GenBank sequences, species designations and strain names are given. The sequences investigated in the present study are marked in blue, red, orange and purple.

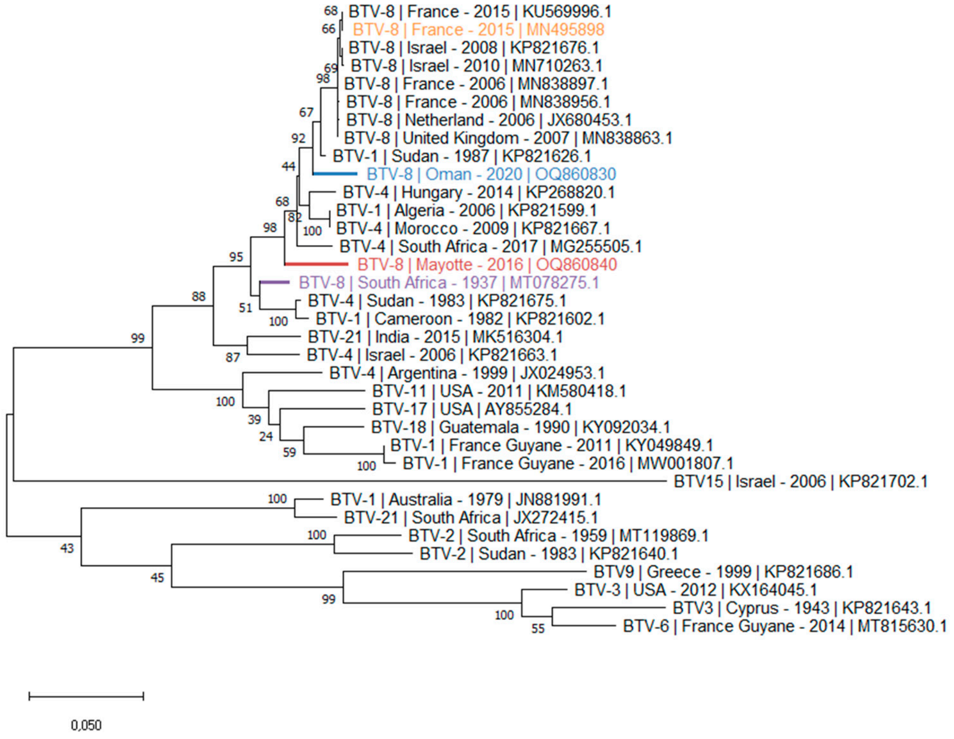

Figure S5: Phylogenetic analysis of BTV S7 sequences. Phylogenetic analysis of S7 sequences of BTV strains using the Maximum Likelihood method and Tamura-Nei model (1000 replicates). This analysis involved 35 nucleotide sequences, there were a total of 1141 positions in the final dataset. In the phylogenetic tree, GenBank sequences, species designations and strain names are given. The sequences investigated in the present study are marked in blue, red, orange and purple.

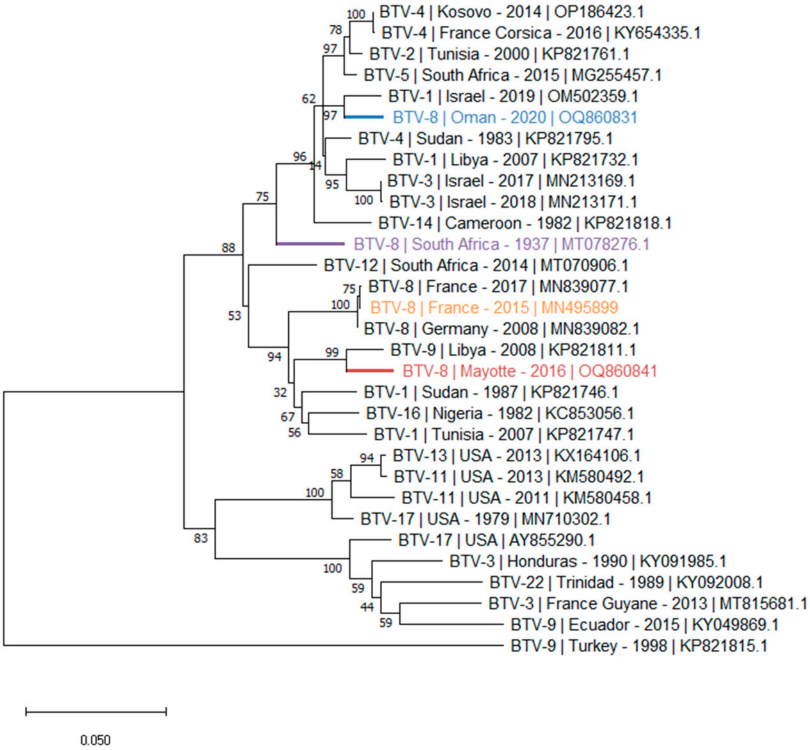

Figure S6: Phylogenetic analysis of BTV S8 sequences. Phylogenetic analysis of S8 sequences of BTV strains using the Maximum Likelihood method and Tamura-Nei model (1000 replicates). This analysis involved 31 nucleotide sequences, there were a total of 1073 positions in the final dataset. In the phylogenetic tree, GenBank sequences, species designations and strain names are given. The sequences investigated in the present study are marked in blue, red, orange and purple.

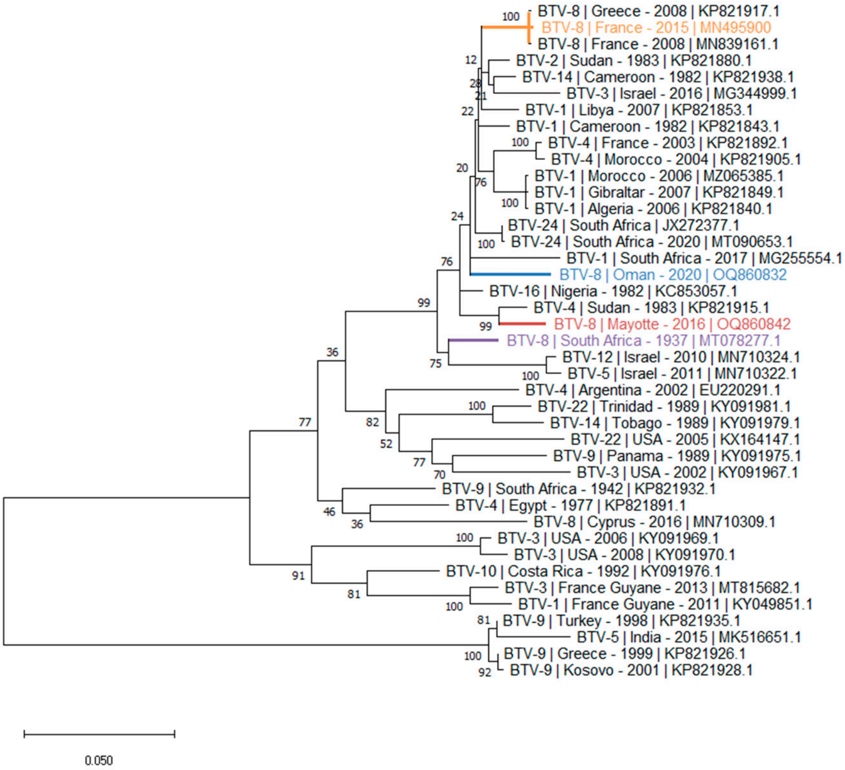

Figure S7: Phylogenetic analysis of BTV S9 sequences. Phylogenetic analysis of S9 sequences of BTV strains using the Maximum Likelihood method and Tamura-Nei model (1000 replicates). This analysis involved 41 nucleotide sequences, there were a total of 1037 positions in the final dataset. In the phylogenetic tree, GenBank sequences, species designations and strain names are given. The sequences investigated in the present study are marked in blue, red, orange and purple.

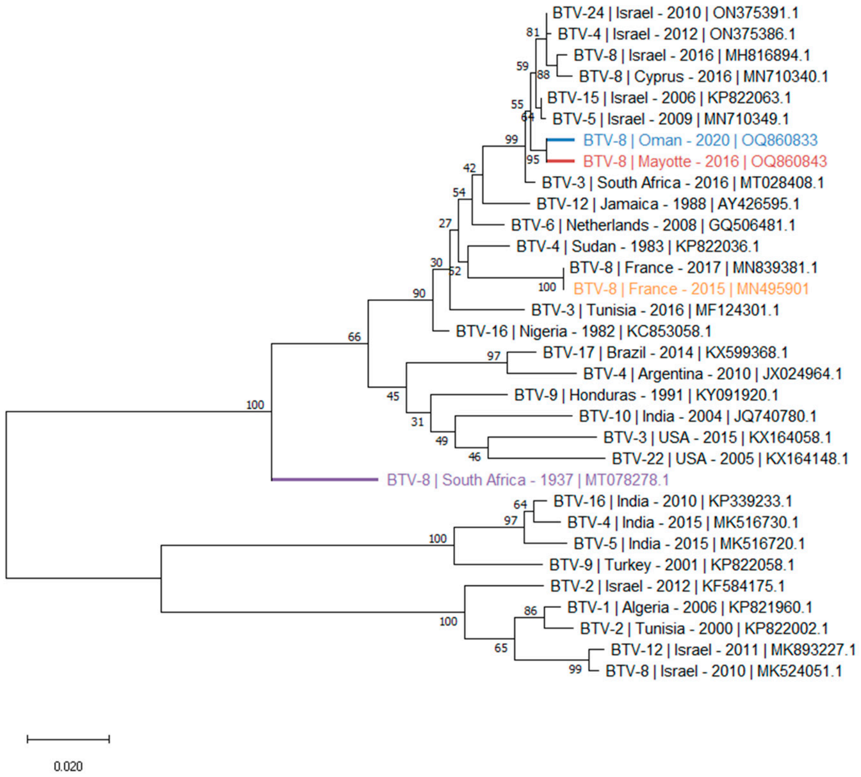

Figure S8: Phylogenetic analysis of BTV S10 sequences. Phylogenetic analysis of S10 sequences of BTV strains using the Maximum Likelihood method and Tamura-Nei model (1000 replicates). This analysis involved 32 nucleotide sequences, there were a total of 1073 positions in the final dataset. In the phylogenetic tree, GenBank sequences, species designations and strain names are given. The sequences investigated in the present study are marked in blue, red, orange and purple.
